# Toward Supportive Decision-Making for Ureteral Stent Removal: Development of a Morphology-Based X-Ray Analysis

**DOI:** 10.3390/bioengineering12101084

**Published:** 2025-10-05

**Authors:** So Hyeon Lee, Young Jae Kim, Tae Young Park, Kwang Gi Kim

**Affiliations:** 1 Department of Biohealth & Medical Engineering, Gachon University, Seongnam 13120, Republic of Korea; l03hyun99@gachon.ac.kr; 2 Gachon Biomedical & Convergence Institute, Gachon University Gil Medical Center, Incheon 21555, Republic of Korea; kimyj10528@gmail.com; 3 Department of Urology, Gil Medical Center, Gachon University College of Medicine, Incheon 21565, Republic of Korea; pty0906@naver.com; 4 Department of Biomedical Engineering, Gil Medical Center, Gachon University College of Medicine, Incheon 21565, Republic of Korea

**Keywords:** ureteral stent, automated image analysis, quantitative morphology, gradient-based method, clinical decision support

## Abstract

Purpose: Timely removal of ureteral stents is critical to prevent complications such as infection, discomfort and stent encrustation or fragmentation, as well as stone formation associated with neglected stents. Current decisions, however, rely heavily on subjective interpretation of postoperative imaging. This study introduces a semi-automated image-processing algorithm that quantitatively evaluates stent morphology, aiming to support objective and reproducible decision-making in minimally invasive urological care. Methods: Two computational approaches were developed to analyze morphological changes in ureteral stents following surgery. The first method employed a vector-based analysis, using the FitLine function to derive unit vectors for each stent segment and calculating inter-vector angles. The second method applied a slope-based analysis, computing gradients between coordinate points to evaluate global straightening of the ureter over time. Results: The vector-angle method did not demonstrate significant temporal changes (*p* = 0.844). In contrast, the slope-based method identified significant ureteral straightening (*p* < 0.05), consistent with clinical observations. These results confirm that slope-based quantitative analysis provides reliable insight into postoperative morphological changes. Conclusions: This study presents an algorithm-based and reproducible imaging analysis method that enhances objectivity in postoperative assessment of ureteral stents. By aligning quantitative image processing with clinical decision support, the approach contributes to precision medicine and addresses the absence of standardized criteria for stent removal.

## 1. Introduction

Waste products in the human body are excreted in urine by the kidneys. Urine is collected in the renal pelvis, a funnel-shaped structure, before being transported to the bladder through the ureter [1]. The ureter functions as a conduit carrying urine from the renal pelvis to the bladder, and ureteral obstruction may occur due to various causes, such as stones, tumors, or strictures [2]. Ureteral strictures can be caused by acquired conditions such as trauma, surgical injury, infection, ureteral stones, and malignant tumors. In addition, congenital abnormalities such as incomplete ureteral muscle formation may impair ureteral peristalsis and result in urinary obstruction [3]. The incidence of urolithiasis has been steadily increasing worldwide, with reported prevalence rates of 7–13% in North America, 5–9% in Europe, and 1–5% in Asia [4,5]. This condition is prone to recurrence and is highly prevalent, resulting in significant treatment costs and the possibility of both acute and chronic complications in patients [6]. When urine cannot be properly excreted because of conditions such as urolithiasis, it can increase the pressure on the kidney, affecting kidney function. Ureteral stenting is one of the management procedures, serving as an important intervention to relieve obstruction and optimize the patient prior to definitive stone treatment [7]. This procedure involves inserting a tube directly into the kidney to facilitate urine flow when the ureter is obstructed or narrowed due to urological disorders, thereby preventing normal urine flow. The ureteral stent assists in the drainage of urine from the kidney to the bladder by acting as an intra-luminal bypass, facilitating urine outflow. The primary purpose of ureteral stents is to maintain urine drainage and to reduce early or late complications associated with urinary tract obstruction [8]. The stent is also used to prevent ureteral obstruction caused by swelling or residual stone fragments following surgical treatment of the kidney or ureteral stones [9]. The timing of ureteral stent removal varies among patients, and the decision is based on clinical parameters and on pre-stent removal radiological evaluation. Radiographs may be performed immediately postoperatively in selected cases when there is doubt about proper positioning or for documentation purposes [10]. To date, no clear standard exists for stent removal, which is typically decided on the basis of the clinician’s judgment [11]. The assessment of stent straightening is subjective and variable among clinicians, and no standardized or quantitative criteria currently exist for evaluating the optimal time for stent removal.

Porto et al. (2025) highlighted through a systematic review and meta-analysis that no consensus exists on the optimal stenting duration, with practices varying widely across institutions [12], while Heidenberg et al. (2023) demonstrated in a randomized trial that even short differences in dwell time (3 vs. 7 days) significantly impacted postoperative symptoms [13]. These findings show the absence of standardized or quantitative criteria for determining the optimal timing of stent removal. In addition, recent advances in AI-based medical imaging have demonstrated the feasibility of automated ureteral stent analysis. Hu et al. (2024) proposed a Mask R-CNN and 3D morphological approach to detect stent encrustation in CT images [14], while Qiu et al. (2023) applied CT radiomics and machine learning to classify stent encrustation with high accuracy [15]. More recently, Wang et al. (2025) developed a deep learning model to automatically measure ureteral length from CT urography images [16]. Together, these studies underscore a clear gap: although encrustation and anatomical dimensions have been quantitatively analyzed, the phenomenon of stent straightening has not.

Clinicians frequently observe that ureteral stents, particularly those placed in tortuous or obstructed ureters, appear more straightened in follow-up X-ray imaging as ureteral decompression progresses over time. Such straightening has been clinically observed, but it has never been quantitatively analyzed or validated. This disconnect between clinical intuition and objective evidence presents a critical research gap. Conventional imaging interpretations rely on qualitative assessment, which lacks reproducibility and cannot capture subtle morphological changes. Thus, there is a growing need for automated, quantitative tools that can integrate seamlessly into clinical workflows.

Therefore, among the various factors considered in determining stent removal, we specifically targeted ureteral straightening and sought to analyze it quantitatively. This approach does not propose straightening as a stand-alone determinant but rather aims to formalize a commonly observed yet previously unvalidated phenomenon, offering an adjunctive imaging marker to complement established clinical criteria.

## 2. Materials and Methods

This study was approved by the Institutional Review Board (IRB) of Gachon University Gil Hospital (approval number: [GFIRB2024-310]) on 22 October 2024. Radiographs and demographic data, including gender, age, body mass index (BMI), stent location, and stent insertion duration, were obtained from 200 patients who underwent ureteral stent insertion surgery at the Gachon University Gil Hospital. The data were accessed for research purposes on 23 October 2024, and as this retrospective study was conducted using de-identified clinical data, the requirement for informed consent was waived by the Institutional Review Board. The first radiograph was acquired immediately after stent insertion and the second radiograph after a clinically determined follow-up period. Initially, radiographic and clinical data were collected from 196 patients. Patients with clearly visible single ureteral stents and at least two radiographs were included. Cases with tandem stents or with poor anatomical clarity were excluded to avoid confounding curvature analysis. After applying these criteria, 173 patients were included in the final analysis. Table 1 provides a detailed overview of the demographic and clinical characteristics of the patients, highlighting variables such as sex, age, BMI classification, stent location, and stent duration. A post hoc power analysis was subsequently performed using G*Power 3.1 based on the paired *t*-test outputs from SPSS Statistics for Windows, Version 20.0 (IBM Corp., Armonk, NY, USA) (two-tailed, α = 0.05). Effect sizes were calculated as Cohen’s d for paired samples, confirming that the sample size was sufficient to detect meaningful differences.

Of the total cohort, 57.8% were male (n = 100) and 42.2% were female (n = 73). Age distribution had the highest representation in the 60–79 age group (45.1%, n = 78), with a mean age of 67.4 ± 5.1 years for this category. The BMI classification showed that 44.5% of patients were obese (≥25; n = 77), with a mean BMI of 28.24 ± 3.03. Stent location data revealed that the left side was most commonly affected (47.98%; n = 83). The stent duration categories demonstrated that 46.24% of patients (n = 80) had a stent duration of ≤30 days, with a mean duration of 22.45 ± 4.82 days for this subgroup.

**Figure 1:**
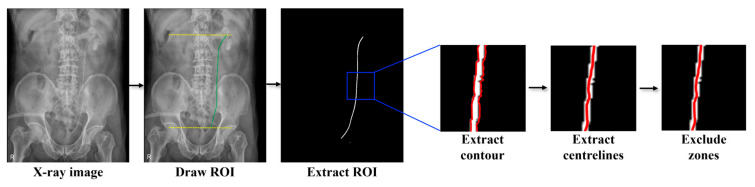
Preprocessing flowchart; The yellow lines represent the vertebral reference lines used to define the region of interest (ROI), the green line indicates the ureteral stent region, and the red lines show the extracted contour, centerline, and the excluded zones at both ends during preprocessing.

Figure 1 shows a flowchart of the labeling and preprocessing process. In X-ray images, the ureteral stent region was designated as the region of interest (ROI) using Aview software (v1.1.44.17-win, Coreline Soft, Seoul, Republic of Korea), based on the start and end points of the ureteral stent relative to the vertebrae of patients who had undergone ureteral stent insertion surgery. To minimize subjectivity, ROI designation was independently cross-checked by two board-certified urologists, thereby reducing inter-observer variability and enhancing reliability. After binarizing the ROI, the contour extraction function provided by the open-source computer vision library (OpenCV, version 3.4.2) [17], an open-source library used in computer vision and image processing applications, was used to extract the outline of the ROI. From the extracted contours, the central values of the coordinates on the same line are calculated to obtain a single curve for the ROI. To eliminate noise from pigtail curvatures at both ends of the stent, the top and bottom 5% were excluded from further analysis. This cutoff was determined through preliminary experiments and repeated discussions with experienced clinicians. Finally, the binarized images from the preprocessing stage were used in both algorithmic studies.

**Figure 2:**
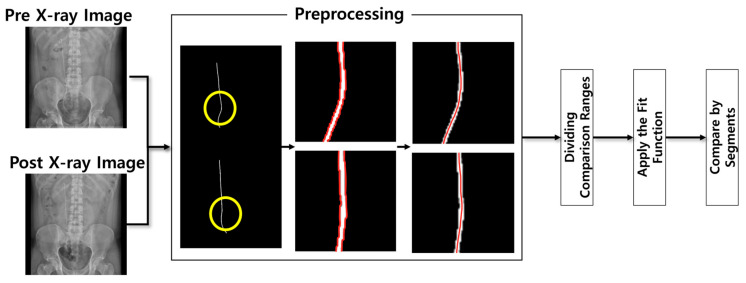
Flowchart of the ureter angle change measurement algorithm; The yellow circles indicate the regions that are magnified and displayed in the subsequent preprocessing panels, and the red lines represent the extracted contour and centerline of the ureteral stent used for comparative angle analysis.

Figure 2 presents a flowchart of Algorithm 1, which was designed to assess the changes in the ureter following ureteral stent insertion. In this algorithm, the Fitline function was applied to the comparison segments, and the angle between the vectors was measured using the unit vectors of the straight line immediately after surgery and at subsequent time points.

The FitLine function extracts a straight line that best fits a given set of points, providing points on the line and a unit vector of the line [18]. The preprocessed ureter was divided into 100 segments along its length, and the FitLine function was applied to the coordinates within each segment to measure the angle of change in the ureter after stent insertion. Specifically, the FitLine function was used on the first (1/100) and subsequent segments (2/100) immediately after surgery to obtain vectors for these comparison segments. To assess the changes across segments, the angle between these two vectors was measured using the formula shown in Equation (1). In this formula, ${vx}_{1}$ and ${vy}_{1}$ represent the vector components of the first segment immediately after surgery, whereas ${vx}_{2}$ and ${vy}_{2}$ represent those of the second segment. Since the acos() function is used to calculate the angle, values between −1 and 1 are required; therefore, any out-of-range values were set to return 0. The values obtained from the acos() function were in radians, and the conversion formula in Equation (3) was used to express ureteral changes in degrees. This algorithm was applied to the ureter immediately after surgery and at a later time point for the same patient to calculate the angle changes in the ureter. These values were averaged to compare the mean angle change immediately after surgery with that after a certain period.

(1) $$\theta =\cos^{-1}\frac{{vx}_{1}{vx}_{2}+{vy}_{1}{vy}_{2}}{\sqrt{{{vx}_{1}}^{2}+{{vy}_{1}}^{2}}\sqrt{{{vy}_{2}}^{2}+{{vy}_{2}}^{2}}}$$

(2) $$=\;{cos}^{-1}\left(\frac{\vec{u}•\vec{v}}{∥\vec{u}∥∥\vec{v}∥}\right)$$

(3) $$degree\;=\frac{radian\times 180}{3.14}$$

**Figure 3:**
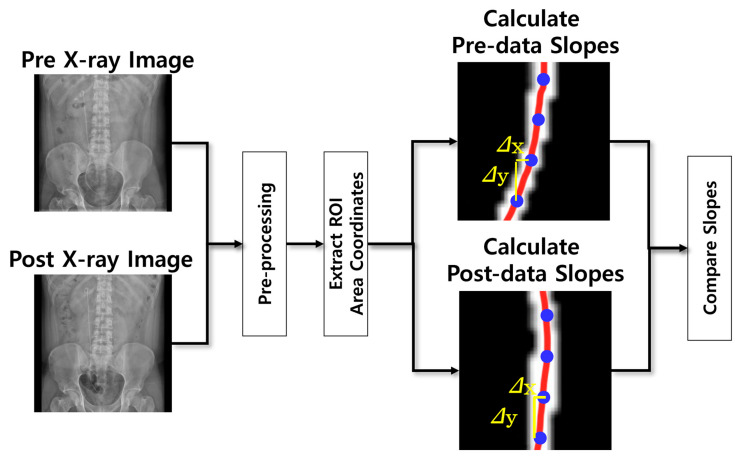
Flowchart of the Ureter Total Slope Change Measurement Algorithm.

Figure 3 presents a flowchart of Algorithm 2, which was designed to assess the changes in the ureter following ureteral stent insertion. In this algorithm, the coordinates of the ureter were extracted from each preprocessed image captured before and after surgery, and the slope values were calculated and compared to evaluate the difference in ureteral curvature immediately after surgery and at later time points.

In the second algorithm, which evaluates ureteral changes by measuring the overall curvature, the slope is determined by extracting the coordinate values corresponding to the ureter from the binarized image obtained through pre-processing. The rate of change expressed in Equation (4) was calculated for all the extracted coordinates, and these values were summed to compare the total curvature of the ureter immediately after stent insertion with that at a later time point. In this equation, x_1_ and y_1_ represent the Nth coordinates, whereas x_2_ and y_2_ represent the N + 1th coordinates

(4) $$gradiant=\frac{y_{2}-y_{1}}{x_{2}-x_{1}}=\frac{∆y}{∆x}$$

## 3. Results

**Figure 4:**
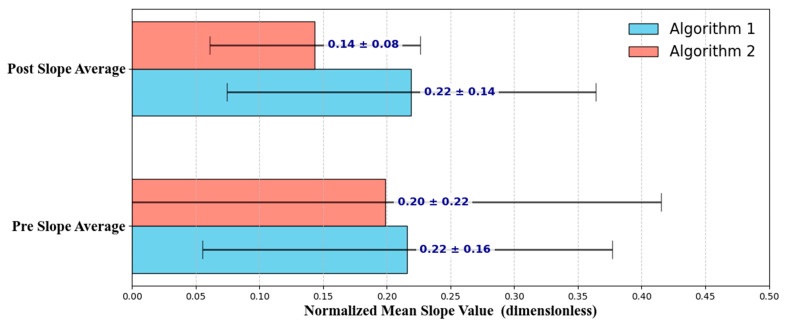
Mean and standard deviation of pre- and post-operation slopes for two algorithms.

A *t*-test is a statistical method suitable for comparing mean differences between groups. The *t*-test was employed in this study to compare population means when the population variance and standard deviation were unknown, using sample variance and standard deviation to analyze the difference in means between the two groups, and the statistical analyses were performed using Microsoft Excel for Microsoft 365 (Microsoft Corp., Redmond, WA, USA) [19]. There are three types of *t*-tests: one-sample, independent two-sample, and paired or dependent sample [20]. In this study, a paired sample *t*-test was applied to compare changes in the ureter of a single patient immediately before and after ureteral stent insertion. A paired-sample *t*-test is used when the groups being compared are the same before and after the experiment, making it suitable for cases where the same individual is analyzed under two different conditions [21]. In this study, the null hypothesis was set to ‘there is no change in the ureter immediately after surgery and after a certain period,’ with the alternative hypothesis being ‘there is a change in the ureter immediately after surgery and after a certain period. The *p*-value was used to assess whether changes occurred in the ureter following ureteral stent insertion. Normality was assessed using the Shapiro–Wilk test. As the assumption of normality was not satisfied, the Wilcoxon signed-rank test was applied in addition to the paired *t*-test. The first algorithm measured changes in the angle of the ureter by segmenting X-ray images taken immediately post-surgery and after a set period. Using the FitLine function, the unit vectors for each segment were extracted, and the angles between these vectors were calculated to analyze ureteral changes; however, no statistically significant differences were found (paired *t*-test: *p* = 0.844; Wilcoxon signed-rank test: *p* = 0.406). This suggests that using the angle difference by segment may not provide sufficient information to accurately reflect the degree of ureteral straightening. The second algorithm measures the overall change in the ureteral slope. The coordinates of the ureter were extracted from the binarized image after pre-processing, and the slope between each coordinate was calculated to compare changes in the slope immediately following surgery and after a certain period. As shown in Figure 4, Algorithm 2 more clearly differentiates between pre- and post-operative ureteral morphology, with reduced variance and stronger group separation. Postoperative normalized slope in Algorithm 2 decreased from 0.20 ± 0.22 to 0.14 ± 0.08, while Algorithm 1 showed no meaningful change. These results suggest that slope-based analysis may serve as a more reliable indicator of stent straightening in follow-up evaluations. A statistically significant difference was observed (paired *t*-test: *p* = 0.002; Wilcoxon signed-rank test: *p* = 0.031), indicating that assessing the overall change in the ureteral slope can more accurately evaluate the degree of straightening after stent insertion. Method 2 demonstrated a difference between the slope of the postoperative ureter immediately following surgery and at a later time.

The bar graphs in Figure 4 compare the normalized mean slope values of the ureter calculated before and after the procedure using the two different algorithms. The blue bars represent the values for Algorithm 1, whereas the red bars represent those for Algorithm 2. The error bars indicate the standard deviation for each group. The pre-procedure mean slope value was 0.22 ± 0.16 for Algorithm 1 and 0.20 ± 0.22 for Algorithm 2, showing no significant difference between the two algorithms. However, the postoperative mean slope value remained nearly unchanged for Algorithm 1 at 0.22 ± 0.14, whereas for Algorithm 2 it exhibited a significant decrease (0.14 ± 0.08), indicating that Algorithm 2 more accurately captured the reduction in ureter slope after the procedure. Algorithm 1, which measures and averages the angle changes by segment, did not show statistically significant differences. In contrast, Algorithm 2, which compares the average slope across the entire ureter length, yielded statistically significant results, as illustrated in the bar graph of Figure 4. These findings suggest that the second algorithm, which measures the overall slope change in the ureter, may serve as a more reliable indicator for determining the appropriate timing for ureteral stent removal. Furthermore, post hoc power analysis supported these findings: Algorithm 2 demonstrated an effect size of d = 0.35 with power ≈ 0.99, confirming that the sample size was sufficient to detect meaningful differences. By contrast, Algorithm 1 showed only a negligible effect (d ≈ 0.02) with very low power (~0.05), indicating that its non-significant result was attributable to the absence of a meaningful effect rather than insufficient sample size.

## 4. Discussion

This study aimed to develop quantitative measurement algorithms that may provide supportive information regarding the timing of ureteral stent removal. Although the timing of stent removal is influenced by various clinical factors, the degree of ureteral straightening has often been assessed subjectively. To address this limitation, our study provides a quantitative and objective measure of straightening as a supportive tool, thereby helping to fill an important clinical gap in ureteral stent management.

Two algorithms were developed and compared in this study to evaluate morphological changes in the ureter over time. The first algorithm analyzes ureteral changes by extracting unit vectors from each segment, measuring the angles between them using the FitLine function to extract segment-wise vectors. In contrast, the second algorithm measures the overall slope change in the ureter by comparing it immediately after surgery with that after a certain period. The *t*-test results of the second algorithm showed statistically significant changes, indicating that it more accurately assesses the degree of stent straightening by evaluating the overall slope change in the ureter. The lack of statistically significant results from the first algorithm may have been due to errors in defining the ROI corresponding to the ureteral stent. Although the ROI was set relative to the patient’s spine, ensuring exact positional consistency was challenging, which could have hindered comparison of the same segments. In addition, inconsistencies in patient posture and imaging conditions during X-ray acquisition may have introduced errors. Moreover, Algorithm 1, which relied on segment-wise angle analysis, failed to show meaningful changes. Because the angle was calculated locally for each small segment, accumulated subtle changes were often averaged out, resulting in insensitivity to gradual global straightening. These factors reduce the accuracy of ureteral angle measurements, highlighting the need for alignment techniques in future research to enable more precise comparisons and analyses. Alignment methods are widely used in medical image analysis and help reduce discrepancies between images, allowing for accurate anatomical comparisons [20]. In addition, the degree of straightening may also be influenced by stent rigidity, baseline anatomic variability, and the persistence of hydronephrosis. These biological and anatomical factors highlight the need for multi-factorial analysis in future research, allowing for a more comprehensive and reliable assessment of ureteral stent morphology.

While clinical decision-making for ureteral stent removal typically involves a combination of factors, such as renal function, infection status, and patient symptoms, the interpretation of follow-up imaging remains an important and widely used element. However, current radiographic assessments, particularly those evaluating the degree of stent straightening, are inherently subjective and lack standardized, quantifiable criteria.

Although it is often implicitly observed in clinical practice that ureteral stents appear more straightened over time as obstruction resolves, this phenomenon has not been previously quantified or systematically analyzed using imaging data. Therefore, this study represents one of the first attempts to objectively formalize such clinical impressions through quantitative radiographic analysis.

The proposed algorithm addresses this gap by providing a method to objectively quantify morphological changes, specifically by measuring ureteral slope across serial radiographs. By translating visual impressions into numerical values, this approach offers more consistent and reproducible imaging interpretation, ultimately enhancing clinical decision-making.

In conclusion, this study introduces a slope-based analysis method for quantitatively assessing ureteral stent straightening over time, addressing the current lack of standardized criteria for removal timing. Compared to conventional visual assessment, the proposed method offers enhanced objectivity and reproducibility. These findings suggest that the technique may be integrated into clinical workflows, potentially guiding real-time decision-making during follow-up and reducing subjectivity in postoperative evaluations. Despite these strengths, this study has limitations. In particular, hydronephrosis grading was not quantitatively assessed, although it was qualitatively considered in the clinical decision-making process. Future work may therefore focus on automating stent detection and alignment, as well as incorporating systematic hydronephrosis grading, to further improve usability in diverse imaging environments.

## Figures and Tables

**Table 1:** Patient demographics and clinical characteristics of ureteral stent insertion cases.

Variable	Category	n	Percent	Mean ± SD
Sex	M	100	57.80%	-
	F	73	42.20%	-
Age	≤20	0	3.30%	0.0 ± 0.0
	21–39	23	13.30%	33.8 ± 5.0
	40–59	64	37.00%	50.1 ± 6.1
	60–79	78	45.10%	67.4 ± 5.1
	≥80	8	4.60%	85.6 ± 3.5
BMI	Underweight (<18.5)	5	2.89%	18.02 ± 0.36
	Normal (18.5–22.9)	36	20.81%	21.27 ± 0.92
	Overweight (23–24.9)	55	31.79%	24.16 ± 0.54
	Obese (≥25)	77	44.51%	28.24 ± 3.03
Stent Location	Left	83	47.98%	-
	Right	71	41.04%	-
	Both	19	10.98%	-
Stent Duration	≤30 days	80	46.24%	22.45 ± 4.82
	31–60 days	78	45.09%	37.04 ± 5.13
	61–120 days	9	5.20%	77.22 ± 13.53
	≥121 days	6	3.47%	290.83 ± 178.33

## Data Availability

The data is not publicly available due to legal and ethical restrictions. However, portions of the data may be available upon reasonable requests after discussion with the corresponding author.
